# Health economics research into supporting carers of people with dementia: A systematic review of outcome measures

**DOI:** 10.1186/1477-7525-10-142

**Published:** 2012-11-26

**Authors:** Carys Jones, Rhiannon Tudor Edwards, Barry Hounsome

**Affiliations:** 1 Centre for Health Economics and Medicines Evaluation, IMSCaR, College of Health and Behavioural Sciences, Bangor University, Dean Street Building, Gwynedd, LL57 1UT, UK

**Keywords:** Carers, Dementia, Quality of life, EQ-5D, Outcome measures

## Abstract

Advisory bodies, such as the National Institute for Health and Clinical Excellence (NICE) in the UK, advocate using preference based instruments to measure the quality of life (QoL) component of the quality-adjusted life year (QALY). Cost per QALY is used to determine cost-effectiveness, and hence funding, of interventions. QALYs allow policy makers to compare the effects of different interventions across different patient groups. Generic measures may not be sensitive enough to fully capture the QoL effects for certain populations, such as carers, so there is a need to consider additional outcome measures, which are preference based where possible to enable cost-effectiveness analysis to be undertaken. This paper reviews outcome measures commonly used in health services research and health economics research involving carers of people with dementia. An electronic database search was conducted in PubMed, Medline, the Cumulative Index to Nursing and Allied Health Literature (CINAHL), PsycINFO, the National Health Service Economic Evaluation Database (NHS EED), Database of Abstracts of Reviews of Effects (DARE) and Health Technology Assessment database. Studies were eligible for inclusion if they included an outcome measure for carers of people with dementia. 2262 articles were identified. 455 articles describing 361 studies remained after exclusion criteria were applied. 228 outcome measures were extracted from the studies. Measures were categorised into 44 burden measures, 43 mastery measures, 61 mood measures, 32 QoL measures, 27 social support and relationships measures and 21 staff competency and morale measures. The choice of instrument has implications on funding decisions; therefore, researchers need to choose appropriate instruments for the population being measured and the type of intervention undertaken. If an instrument is not sensitive enough to detect changes in certain populations, the effect of an intervention may be underestimated, and hence interventions which may appear to be beneficial to participants are not deemed cost-effective and are not funded. If this is the case, it is essential that additional outcome measures which detect changes in broader QoL are included, whilst still retaining preference based utility measures such as EQ-5D to allow QALY calculation for comparability with other interventions.

## Background

In the UK, the government faces an increasing challenge to meet the growing demands on the healthcare system. Despite increased public expectations of treatment availability, an ageing population and higher levels of chronic disease, the government is aiming to achieve efficiency savings of £20 billion in the National Health Service’s (NHS) budget by 2014 [1]. Savings are to be made through focusing on quality, innovation, productivity and prevention. Treatments offered on the NHS must be clinically effective and cost-effective, as assessed by the National Institute for Health and Clinical Excellence (NICE). The NICE guide to technology appraisal [2] states that cost-effectiveness should be reported in Quality Adjusted Life Years (QALYs), a measure combining length of time with quality of life (QoL). Therefore, the choice of instrument used to measure QoL is important, as the resulting QALY calculations determine whether a treatment is cost-effective and hence potentially funded. The issue of NICE cost-effectiveness funding thresholds may only be applicable to the UK; however, the methodological issue of measuring and valuing carer benefits has international application.

Dementia places a large burden on the economy, with costs incurred by the health care sector, social care sector and informal carers [3]. The largest proportion of the cost (55%) is incurred by informal carers looking after a friend or relative, and is indicative of the burden faced by carers. Carer burden can predict institutionalisation of the person with dementia [4,5]; therefore evidence of effective methods to support carers in their role needs to be established to delay institutionalisation and the associated costs. Burden can affect QoL through decreased mental wellbeing caused by stress and worry, and also the opportunity cost of reduced time for leisure activities and self-care [6].

The need to use appropriate outcome measures in health economics research has been recognised [7-9]. Interventions involving people with dementia and their carers may be complex with multiple objectives; therefore it is necessary to consider multiple outcome measures. Focusing on one attribute, such as QoL, may lead to other benefits being overlooked. Moniz-Cook et al. [10] argued that a more cohesive approach to outcome measurement in dementia care research will lead to a more robust evidence base. Health economists require preference based utility measures for calculating QALYs. However, restricting benefit measurement to health-related outcomes in carer research places a patient identity on the carer, which may not be appropriate [11]. This article aims to address the question ‘what outcome measures are used most frequently in interventions involving carers of people with dementia, and how useful are these measures for economic evaluation?’

## Methods

A systematic literature search of electronic databases was conducted on 1st March, 2012. PRISMA reporting principles were used as guidance [12]. PubMed (1946–2012), Medline (1950–2012), the Cumulative Index to Nursing and Allied Health Literature (CINAHL) (1981–2012), PsycINFO (1806–2012), and the NHS Centre for Reviews and Dissemination (containing the National Health Service Economic Evaluation Database (NHS EED), Database of Abstracts of Reviews of Effects (DARE) and Health Technology Assessment database) (1960–2012) were searched. Titles, keywords and abstracts were searched for the terms caregiver, randomized controlled trials and dementia or Alzheimer’s disease using MeSH terms where possible. The search strategy for each database is presented in Additional file 1: Appendix 1. No restriction on publication year was set. Study eligibility was based on initial screening of title and abstract. Articles passing initial screening were retrieved for further review.

Studies were considered if they reported an intervention with outcome measures for carers of people with dementia. Carers could be paid workers or informal carers, such as friends and family members. We included outcomes for paid carers to get a broader indication of which aspects of health and social care provision are typically measured. No gender, age or nationality restrictions were applied. The person being cared for could be living in residential care, a medical facility or the community.

Carer outcome measures were extracted and categorised. The categories used in Moniz-Cook et al. [10] were a starting point: burden, mood, quality of life and staff competency and morale. Two additional categories were developed after reviewing the data: mastery and social support and relationships.

## Results

**Figure 1:**
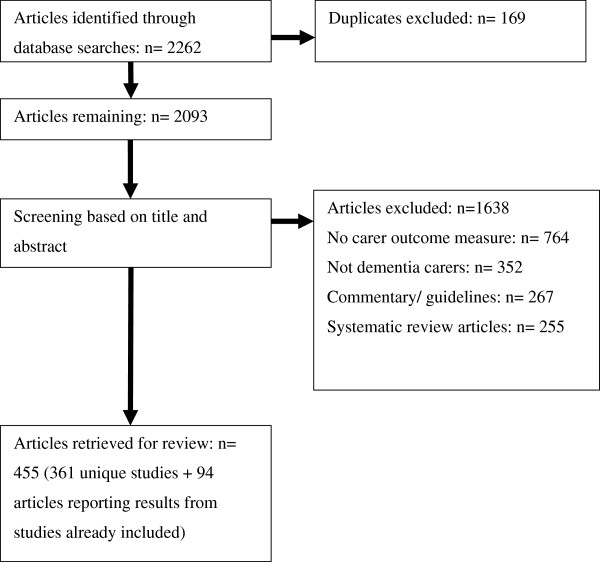
Flow of articles retrieved through electronic searches.

2262 records were retrieved, 2093 articles remained after duplicates were removed (Figure 1). After screening titles and abstracts, 1638 articles were excluded. Exclusion reasons included no carer outcome measure (764 articles), the population not consisting of dementia carers (352 articles), commentary articles or clinical practice guidelines (267 articles) and systematic review articles (255 articles). 455 articles reporting on 361 studies remained. 228 outcome measures were extracted. A full list of extracted outcome measures, the number of studies they appeared in and their earliest and most recent year is in Additional file 2: Appendix 2. Table 1 presents key properties of outcome measures appearing in four or more studies (1% of included studies). Table 2 shows the change in composition of carer outcome measures used over the years.

**Table 1:** Properties of the most frequently used outcome measures

**Category**	**Outcome measure**	**Number of studies**	**Publication year**	**Region of development**	**Number of items**	**Number of levels per item**	**Dementia specific?**
Burden	Zarit Burden Interview	76 (21.1%)	1983	USA	22	5	Yes
	Revised Memory and Behavior Problems Checklist (RMBPC)	44 (12.2%)	1992	USA	24	6	Yes
	Relatives Stress Scale	13 (3.6%)	1982	UK	15	5	Yes
	Novak Caregiver Burden Inventory	11 (3.0%)	1989	Canada	24	4	Yes
	Screen for Caregiver burden	11 (3.0%)	1991	USA	25	5	Yes
	Perceived Stress Scale	11 (3.0%)	1983	USA	14	5	No
	(Revised) Caregiving Burden Scale	6 (1.7%)	1994	Netherlands	13	5	Yes
	Caregiver Stress Scale	4 (1.1%)	1990	USA	15	3-5	Yes
Mastery	Sense of Competence Questionnaire	12 (3.3%)	1996	Netherlands	27	2-5	Yes
	Brief Coping Orientation for Problems Experienced (COPE)	6 (1.7%)	1997	USA	28	4	No
	Ways of coping scale	6 (1.7%)	1985	USA	64	2	No
	Revised Scale for Caregiving Self Efficacy	6 (1.7%)	1999	USA	19	Rated 0-100	No
Mood	Center for Epidemiologic Studies Depression Scale (CES-D)	57 (15.8%)	1977	USA	20	4	No
	General Health Questionnaire (GHQ)^1^	31 (8.6%)	1978	UK	28	4	No
	Neuropsychiatric Inventory- Carer Distress (NPI-D)	30 (8.3%)	1998	USA	12	6	Yes
	Geriatric Depression Scale	19 (5.3%)	1982	USA	30	2	No
	Beck Depression Inventory (BDI)	17 (4.7%)	1961	USA	21	4	No
	Neuropsychiatric Inventory Questionnaire (NPI-Q)	12 (3.3%)	2000	USA	12	6	Yes
	Brief Symptom Inventory	8 (2.2%)	1983	USA	53	5	No
	Hamilton Depression Scale	8 (2.2%)	1960	UK	17	3-5	No
	Pittsburgh Sleep Quality Index	8 (2.2%)	1989	USA	24	1-4	No
	Hospital Anxiety and Depression Scale	7 (1.9%)	1983	UK	14	4	No
	State-Trait Anxiety Inventory	7 (1.9%)	1970	USA	20	4	No
	Positive and Negative Affect Scale (PANAS)	5 (1.4%)	1988	USA	20	5	No
	Hopkins Symptoms Checklist	4 (1.1%)	1974	USA	58	4	No
	Positive Aspects of Caregiving	4 (1.1%)	2004	USA	9	5	Yes
Quality of life	Short Form-36 (SF-36)	32 (8.8%)	1988	USA	36	2-6	No
	EuroQoL (EQ-5D)	18 (5.0%)	1990	Europe	5	3	No
	World Health Organization Quality of Life-Bref (WHOQOL-BREF)	8 (2.2%)	1996	Global	26	5	No
	Health Utilities Index Mark^2^	4 (1.1%)	1990	Canada	8	5-6	No
Social support and relationships	Social Support Questionnaire	7 (1.8%)	1983	USA	27	6	No
	Stokes Social Support network List	4 (1.1%)	1983	USA	N/A Matrix	N/A	No
Staff competency and morale	Maslach Burnout Inventory	10 (2.6%)	1981	USA	22	3-7	No
	Approaches to Dementia questionnaire	4 (1.1%)	2000	UK	19	5	Yes

^1^ Information given for the 28 item version (GHQ-28).

^2^ Information given for the Health Utilities Index Mark III (HUI3).

**Table 2:** Composition of outcome measures across the years

		**Number of measures used**, **percentage composition for the time period**					
**Years**	**Number of included papers**	**Burden**	**Mastery**	**Mood**	**QoL**	**Social support and relationships**	**Staff competency and morale**
1985-89	5	2 (12%)	1 (6%)	11 (65%)	3 (18%)	0	0
1990-94	10	8 (42%)	1 (5%)	7 (37%)	1 (5%)	0	2 (11%)
1995-99	33	27 (36%)	12 (16%)	30 (40%)	1 (1%)	4 (5%)	1 (1%)
2000-04	86	68 (32%)	31 (15%)	69 (33%)	23 (11%)	16 (8%)	4 (2%)
2005-09	148	99 (26%)	60 (16%)	131 (34%)	44(11%)	33 (9%)	19 (5%)
2010-12	79	50 (21%)	40 (17%)	67 (29%)	38 (16%)	24 (10%)	14 (6%)

### Burden measures

The 44 measures in this category consisted of burden, stress and strain. Burden was the second most popular category of measure used in dementia carer research. The Zarit Burden Interview (ZBI) [13] was most popular, appearing in 76 studies (21%). The ZBI is dementia specific, originally a 29-item instrument but also available as a shorter 12-item version [14]. Domains of the ZBI cover physical health, psychological well-being, finances, social life, and relationship with the person with dementia. The earliest paper retrieved which included the ZBI was published in 1994; the ZBI is still used currently. The Revised Memory and Behavior Problem Checklist (RMBPC) [15] was the second most popular measure, appearing in 44 studies (12%). It is also dementia specific and contains 24 items adapted from the Memory and Behavior Problem Checklist (MBPC) [16]. The MBPC assesses the frequency and severity of problems exhibited by a person with dementia and their carer’s reaction to these problems. As with the ZBI, the RMBPC has also been in use since 1994 and is still used today.

### Mastery

Forty-three measures encompassing the family carer’s coping, self-efficacy and competence were extracted. As can be seen in Table 2, mastery measures were infrequently used in earlier studies. Currently, mastery measures account for 17% of the outcome measures included in dementia carer research. The Sense of Competence Questionnaire (SCQ) [17] was most popular, appearing in 12 studies (3%) since the year 2000. The SCQ was developed to measure the ability of carers to cope with looking after people with dementia living at home. Three domains are covered: satisfaction with the person receiving care, satisfaction with one’s own performance as a carer and the impact of caring on the personal life of the carer.

### Mood

Mood measures were included the most frequently, and currently account for almost one third of dementia carer measures included. Sixty-one mood measures covering anxiety, depression, sleep and general mental well-being were extracted. The Center for Epidemiologic Studies Depression Scale (CES-D) [18] was the most frequently used measure, appearing from 1989 onwards. CES-D was followed in frequency by the General Health Questionnaire (GHQ) [19] and the Neuropsychiatric Inventory-Distress (NPI-D) [20]. The NPI-D primarily assesses the frequency and severity of behavioural disturbances occurring in people with dementia, but also asks carers to rate their reaction to the behaviours. The NPI-D is one of the more recently developed mood measures, first appearing in the year 2000.The next most popular measures were the Geriatric Depression Scale [21] which was developed for use in an elderly population, the Beck Depression Inventory (BDI) [22] and the Neuropsychiatric-Questionnaire (NPI-Q) [23], a version of the NPI-D suitable for use in a clinical setting which has appeared in publications from 2006 onwards.

### Quality of life measures

Thirty-two QoL measures were identified. While QoL measure inclusion has increased over the years, only 16% of included outcome measures are currently for QoL. Four outcome measures were used most frequently: the Short Form-36 (SF-36) [24], the EuroQoL (EQ-5D) [25]; the World Health Organization Quality of Life-brief (WHOQOL-BREF) [26] and the Health Utilities Index (HUI) [27]. The SF-36 and EQ-5D appeared in publication from 2001 onwards, while the WHOQOL-BREF is a more recent, appearing 2007 onwards.

The SF-36 evolved from the RAND Health Insurance Experiment, a 15 year study of American health policy; and the Medical Outcome Study of patients with chronic illnesses [24]. The SF-6D was subsequently developed; enabling preference based utility scores and QALYs to be calculated from the SF-36 or SF-12 [28,29]. While it is possible to use the SF-6D directly in a study, developers recommend using the SF-36 or SF-12 and then translating results into the SF-6D. The six domains of the SF-6D are physical functioning, role limitation, social functioning, pain, mental health and vitality.

The EQ-5D was developed in Europe and consists of a questionnaire (EQ-5D) and a visual analogue scale (EQ-VAS). The EQ-5D comprises five domains: mobility; self care; usual activities; pain and discomfort; and anxiety and depression. A scoring algorithm converts responses into an index score which can be used to calculate a QALY. On the EQ-VAS, respondents are presented with a thermometer with markings representing the worst and best imaginable health state. Respondents are asked to draw a line to mark the level they would describe their health as being. While the scoring of the EQ-5D is preference based, the EQ-VAS is not.

The WHOQOL-BREF is derived from the WHOQOL-100, an instrument developed by a global research team and intended to be applicable cross-culturally [26]. The domains of the WHOQOL-BREF can be broadly categorised into physical health, psychological wellbeing, social relationships and the environment. Preference based utility scores are not available for either the WHOQOL-BREF or WHOQOL-100.

The Health Utilities Index has two main versions: the HUI2 used with children, and the HUI3 used with adults. The HUI3 has eight domains: vision, hearing, speech, ambulation, dexterity, emotion, cognition and pain [27].

### Social support and relationships

The earliest published use of a social support or relationship measure was in 1999. Twenty-seven measures were identified in this category. Only the Social Support Questionnaire [30] and the Stokes Social Support Network List [31] were used consistently, neither was developed for dementia carers. The Social Support Questionnaire assesses the respondent’s perceived number of social support contacts and their satisfaction with the social support available. The Stokes Social Support Network List asks respondents to list people they have contact with on a regular basis and whether or not they are relatives. The respondent’s social network size and composition is then determined. The Stokes Social Support Network List is a recent measure, appearing in publications dated 2006–2010.

### Staff competency and morale

Staff competency and morale measures were included from 1994 onwards. Twenty-one measures were identified. Only two questionnaires were used in four or more studies; the Maslach Burnout Inventory [32] and the Approaches to Dementia Questionnaire [33]. Burnout is described as the emotional exhaustion and cynicism experienced by staff involved with people-facing roles [32], and the consequences of burnout are low quality of care, low morale and high staff turnover. The Approaches to Dementia Questionnaire assesses the carer’s attitude towards the care recipient.

## Discussion

The key to selecting appropriate outcome measures is defining what an intervention targets, and therefore what a measure has to be able to capture. As can be seen in Table 2, the composition of measures included in dementia carer research has changed over time. In earlier years, mood measures were the most prevalent. While this is still true of current research, the gap between use of mood and burden measures has narrowed. Measures capturing social support and relationships are more commonly used now.

Whichever instrument is used, NICE prefers results to be converted into a QALY to allow comparisons across different illnesses and interventions [2]. In order to satisfy QALY methodology, quality weights must be based on preferences and anchored on an interval scale which contains full health and death points [34]. Preference-based generic instruments, such as the EQ-5D are preferred; however, ‘*when EQ5D utility data are not available*, *direct valuations of descriptions of health states based on standardised and validated HRQL measures included in the relevant clinical trial*(*s*) *may be submitted*. *In these cases*, *the valuation of descriptions should use the time tradeoff method in a representative sample of the UK population*, *with* ‘*full health*’ *as the upper anchor*, *to retain methodological consistency with the methods used to value the EQ5D*’ [2]. Validity of the instrument selected is important for results to be meaningful. The most popular measures in the QoL category have been validated with members of the general population.

The aggregation of carer and patient QALYs is rarely undertaken; however, one trial of befriending for carers of people with dementia presented the incremental cost-effectiveness ratio (ICER), as calculated with the EQ-5D for the QALY component, for both the carer alone and the carer and person with dementia combined [35]. The intervention was not cost-effective when the ICER was calculated for the carer alone, but became cost-effective when the spill-over effects on the person with dementia were incorporated. Aggregation of QALYs needs to be undertaken cautiously, with the information used to calculate resulting ICERs explicitly stated to allow for comparisons with interventions where QALYs have not been aggregated.

Out of the most popular instruments in the QoL category, only weights for the EQ-5D were derived using the time trade-off method. The SF-6D and HUI3 were valued using a visual analogue scale and standard gamble; the WHOQOL-BREF does not have preference based scoring. Three possible explanations for differences in health state valuations between measures have been put forward: coverage of descriptive systems, sensitivity of dimensions and valuation methods [36]. Instruments which describe more health states will pick up smaller changes in health status and are more appropriate for research where smaller health gains are expected to be made [37], such as research involving carers. The HUI3 can describe 972,000 health states; the SF-6D either 7,500 or 18,000 depending on the version, while the EQ-5D only describes 243 health states. A ‘ceiling effect’, where higher health states are chosen more frequently, is known to be a feature of the EQ-5D. In contrast, the SF-6D appears to have a ‘floor effect’, with responses clustered at the lower end of the scale. The floor effect is amplified in population groups with more physical health problems, so may not be an issue when conducting research with carers of people with dementia. This is because although many carers do have health issues, one may assume that they already have reasonable physical health to be able to cope with the physical aspects of caring.

The World Health Organisation defines health as ‘*a state of complete physical*, *mental and social wellbeing and not merely the absence of disease or infirmity*’; a definition unchanged since 1948 [38]. Furthermore, the seven determinants of health are suggested as: income and social status, education, physical environment, social support networks, genetics, health services and gender [39]. This reinforces the idea that we need to go beyond physical health measurement, and consider other attributes affecting QoL. This is particularly relevant for dementia carers, as research is primarily aimed at relieving burden rather than improving physical health.

While the EQ-5D covers physical domains well there is only one question on mental well-being. Due to the dominance of physical domains, it is not particularly sensitive to changes in carers of people with dementia, who might not see changes in their physical health over time though their QoL is still affected. This issue was raised by Al-Janabi et al. [11], who posed that measuring health related outcomes for carers places a ‘patient’ identity on carers. In a cross-sectional study involving carers of people with dementia completing the HUI2, Neumann et al. [40] found that the stage of Alzheimer’s Disease was a negative predictor of patient utility (as reported by carers completing the HUI2 as a proxy); however, the utility that carers reported for themselves was insensitive to the stage of the care recipients dementia. For research involving carers of people with dementia it may be necessary to include additional outcome measures alongside a generic primary outcome measure for cost-effectiveness analysis.

It has been found that disease specific instruments are better at detecting QoL changes than generic instruments [41]. The main advantage of disease specific instruments is that they are sensitive to changes associated with the disease in question; therefore studies do not need a large sample size. A disadvantage is that co-morbidities may be overlooked; by focusing on QoL changes associated with one particular illness, separate health issues are ignored. As people with dementia and their carers tend to be older, co-morbidities and side effects are particularly relevant. Disease specific instruments are typically focused on the person with the illness; therefore using a population group measure may be more appropriate for carers. Population specific measures cover a broader range of domains than disease specific instruments, with the additional benefit of being more sensitive than a generic instrument. This review found that the most popular instruments in the burden category were developed specifically to measure burden in dementia carers, combining disease specific with population specific domains.

This review found 29 studies which included details of costs; however, most of these were only partial economic evaluations which provided cost-outcome descriptions. Where cost-effectiveness analyses had been performed the unit of effect was typically time e.g. cost per additional year that the person with dementia lived at home, cost per reduction in hours spent on care tasks per day. Cost-utility analysis was included in 3 studies [35,42,43]; the outcome measures used were the EQ-5D, HUI2 and the Caregiver Quality of Life Instrument. All three measures are suitable for QALY calculations. The study that included the cost-utility analysis using the HUI2 [42] aggregated carer and patient QALYs, which as mentioned above is not consistent with traditional QALY methodology. 9 of the studies listing costs were protocols, 7 planned to conduct cost-utility analysis using the EQ-5D and 2 planned to conduct cost-utility analysis using the SF-12 or SF-36.

Overall, burden and mood measures were the most frequently used. The earliest article retrieved from the searches was published in 1987 and included 4 mood measures and 1 QoL measure. Outcome measures in the mood category covered a broad range of symptoms from overall mental well-being, anxiety, depression and sleep quality. A variety of social support measures were used; the two most frequently used measures were not specific to dementia carers. Social support measures have grown in popularity but are still not as frequently used as burden, mastery, mood or QoL measures. The least frequently used category of measure was the staff competency and morale category. A large number of unspecified measures were found, mainly due to poor reporting of study methods precluding the authors of this review being able to identify the measure used. The increased use of guidelines such as CONSORT [44], has improved the quality of reporting of trials in recent years.

## Future directions

The ICECAP index of capability has been developed to measure attributes of QoL rather than influences on QoL e.g. health [45]. The theory is that QoL does not decrease due to poorer health, but instead decreases through limitations in what one can do as a result of poor health i.e. individuals value the activities that they can undertake rather than health itself. In this sense, instruments such as the EQ-5D are only a proxy measure for QoL rather than a direct measure [46]. Two versions of the ICECAP are available: the ICECAP-O, suitable for ages 65+; and the ICECAP-A, suitable for ages 18+. The domains of the ICECAP-O are: love and friendship; thinking about the future; doing things that make you feel valued; enjoyment and pleasure and independence. These domains were developed to measure capability in older members of the general population [47] and have a certain degree of overlap with the categories of burden, mastery, mood, quality of life, and social support and relationships. The domains of the ICECAP-A are similar: security; loved and friendship; independence; achievement and enjoyment and pleasure. Currently, an algorithm to convert ICECAP scores into a QALY is not yet available. One way around this is to perform a mapping exercise of ICECAP scores onto EQ-5D scores. To be valid this would require considerable time and financial resources to construct the necessary data set.

The capability framework has also led to the development of the Adult Social Care Outcomes Toolkit (ASCOT) [48], an instrument to measure social care-related QoL. While the ASCOT does not specifically measure carer well-being, it is a step towards acknowledging the importance of the care environment that a person is living in. Domains of the instrument include: control over daily life; personal cleanliness and comfort; food and drink; personal safety; social participation and involvement; occupation; accommodation cleanliness and comfort; and dignity. While the domains are similar the ICECAP, the advantage that the ASCOT tool has is that it is a preference-based measure with scoring reflecting preferences of the general population [49].

## Conclusion

Few studies currently incorporate economic evaluations alongside clinical trials as routine practice. The choice of instrument used to measure QoL has implications for whether or not a treatment is considered cost-effective and potentially funded. Health economists need to choose instruments appropriate for the population and expected outcomes. Researchers need to consider ease of administration and clarity of instrument to ensure as many participants as possible complete questionnaires. For carers of people with dementia, available time is already restricted so there is a need to avoid overburdening participants with lengthy questionnaires. If an instrument is not sensitive enough to detect changes in QoL for carers of people with dementia, the effect of an intervention which may appear to be beneficial to participants are underestimated. Use of capability based instruments such as the ICECAP and ASCOT should enable decision-makers to compare the value of health and social services that may improve the QoL of an individual without necessarily improving their health, to interventions that impact on both health and QoL [47].

## Abbreviations

ASCOT: Adult social care outcomes toolkit; CES-D: Center for epidemiologic studies depression scale; EQ-5D: Euroqol 5 dimensions; EQ-VAS: Euroqol visual analogue scale; GHQ: General health questionnaire; HUI: Health utilities index; ICECAP-O: Icepop capability measure for older people; ICECAP-A: Icepop capability measure for adults; NHS: National health service; NICE: National institute for health and clinical excellence; NPI-D: Neuropsychiatric inventory-distress; QALY: Quality-adjusted life year; QoL: Quality of life; RMBPC: Revised memory and behavior problem checklist; SCQ: Sense of competence questionnaire; SF-36: Medical outcomes study 36-item short form health survey; WHOQOL-BREF: World health organization quality of life- brief; ZBI: Zarit burden interview.

## Competing interests

The authors declare that they have no competing interests.

## Authors’ contributions

CJ contributed to the concept and design of the study, conducted the systematic review, extracted outcome measures, and drafted the manuscript. RTE and BH contributed to the concept and design of the study and made critical revisions to the manuscript for important intellectual content. All authors read and approved the final manuscript.

## Supplementary Material

Additional file 1 Appendix 1: Search strategy. Click here for file

Additional file 2 Appendix 2: Full list of carer outcome measures extracted. Click here for file
