# Supplementary material for: Health economics research into supporting carers of people with dementia: A systematic review of outcome measures
Source: Health Qual Life Outcomes. 2012 Nov 26;10:142. doi: 10.1186/1477-7525-10-142 (PMC3541129; doi:10.1186/1477-7525-10-142)
Supplement: Additional file 1 — Appendix 1: Search strategy. [file 1477-7525-10-142-S1.docx]

Appendix 1: Search strategy

Pubmed/Medline: 1759 results

(("caregivers"[MeSH Terms] OR "care*"[title] OR "care*"[text]) AND ("dementia"[mesh terms] or "dementia"[title] or "dementia"[text] or "Alzheimer Disease"[Mesh terms] or "alzheimer*"[title] or alzheimer*[text]) AND ("clinical trial"[publication type] or "clinical trials as topic"[mesh terms] or "Randomized controlled trial"[text] or "randomized controlled trial"[title] or “clinical trial”[title] or “clinical trial”[text]))

CINAHL 218 results

((caregivers) and (“randomized controlled trials” or "clinical trials") and (dementia or “Alzheimer’s disease”))

http://0-search.ebscohost.com.unicat.bangor.ac.uk/login.aspx?direct=true&db=c8h&bquery=((caregivers)+AND+(%e2%80%9crandomized+controlled+trials%e2%80%9d+OR+%22clinical+trials%22)+AND+(dementia+OR+%e2%80%9cAlzheimer%e2%80%99s+disease%e2%80%9d))&cli0=LA1&clv0=Y&type=0&site=ehost-live

Centre for Reviews and Dissemination (NHS EED/ HTA/ DARE) 92 results

(caregiver*) or (carer*)

(dementia) or (Alzheimer*)

(clinical trial) or (trial)

#1 AND #2 AND #3

PsycINFO 193 results

((Title:trial) OR (Subject:trial) OR (FullText:trial)) AND ((Title:dementia) OR (Subject:dementia) OR (FullText:dementia) OR (Title:alzheimer*) OR (Subject:alzheimer*) OR (FullText:alzheimer*)) AND ((Title:carer*) OR (Subject:carer*) OR (FullText:carer*) OR (Title:caregiver*) OR (Subject:caregiver*) OR (FullText:caregiver*))
