# Supplementary material for: Health economics research into supporting carers of people with dementia: A systematic review of outcome measures
Source: Health Qual Life Outcomes. 2012 Nov 26;10:142. doi: 10.1186/1477-7525-10-142 (PMC3541129; doi:10.1186/1477-7525-10-142)
Supplement: Additional file 2 — Appendix 2: Full list of carer outcome measures extracted. [file 1477-7525-10-142-S2.docx]

Appendix 2: Full list of carer outcome measures extracted

| Category of measure | Instrument | # of studies | % of studies | Earliest date | Latest date |
| --- | --- | --- | --- | --- | --- |
| Burden | Zarit Burden Interview | 76 | 21.1% | 1994 | 2012 |
|  | Revised Memory and Behavior Problems Checklist (RMBPC) | 44 | 12.2% | 1994 | 2012 |
|  | Unspecified burden, behavioural problems and affect measure | 37 | 10.2% | - | - |
|  | Relatives Stress Scale | 13 | 3.6% | 1998 | 2011 |
|  | Novak Caregiver Burden Inventory | 11 | 3.0% | 2005 | 2011 |
|  | Perceived Stress Scale | 11 | 3.0% | 1996 | 2010 |
|  | Screen for Caregiver burden | 11 | 3.0% | 2000 | 2009 |
|  | (Revised) Caregiver Burden Scale | 6 | 1.7% | 1991 | 2009 |
|  | Caregiver Stress Scale | 4 | 1.1% | 2003 | 2007 |
|  | Lawton Caregiver Appraisal Measure | 3 | 0.8% | 1999 | 2010 |
|  | Unspecified stress measure | 3 | 0.8% | - | - |
|  | Bradburn Affect Scale | 2 | 0.6% | 1989 | 2003 |
|  | Burden Scale for Family caregivers (Grassel) | 2 | 0.6% | 2009 | 2010 |
|  | Carer Strain Index | 2 | 0.6% | 2003 | 2006 |
|  | Decisional Conflict scale | 2 | 0.6% | 2005 | 2011 |
|  | Family Caregiver Conflict scale | 2 | 0.6% | 2010 | 2011 |
|  | Gilleard Strain Scale | 2 | 0.6% | 1999 | 2002 |
|  | Modified Conflict Tactics Scale | 2 | 0.6% | 2006 | 2010 |
|  | Nursing home hassles scale | 2 | 0.6% | 2007 | 2011 |
|  | Apparent Affect rating scale | 1 | 0.3% | 2009 | 2009 |
|  | Behavior Management Scale–Revised | 1 | 0.3% | 2007 | 2007 |
|  | Burnout Measure | 1 | 0.3% | 1994 | 1994 |
|  | Caregiver Assessment of Functional Dependence and Caregiver Upset | 1 | 0.3% | 2003 | 2003 |
|  | Caregiver Bathing Behavior Rating Scale | 1 | 0.3% | 2006 | 2006 |
|  | Caregiver Burden caused by Behavioral and Psychological Symptoms of Dementia (CBBD) | 1 | 0.3% | 2007 | 2007 |
|  | Caregiver Strain (Rose) | 1 | 0.3% | 2003 | 2003 |
|  | Caregiver stress inventory | 1 | 0.3% | 2011 | 2011 |
|  | Caregiving Hassle Scale | 1 | 0.3% | 2001 | 2001 |
|  | Carers Assessment of Difficulties Index (CADI) | 1 | 0.3% | 2008 | 2008 |
|  | Cook-Medley Hostility Scale | 1 | 0.3% | 2010 | 2010 |
|  | Hassles During Bathing Scale | 1 | 0.3% | 2006 | 2006 |
|  | Home Care scale | 1 | 0.3% | 2005 | 2005 |
|  | Interpersonal Conflict Scale | 1 | 0.3% | 2007 | 2007 |
|  | Minimum Data Set for Home Care (MDS-HC) | 1 | 0.3% | 2008 | 2008 |
|  | Montgomery Borgotta Caregiver Burden scale | 1 | 0.3% | 2011 | 2011 |
|  | Multiple Affect Adjective Checklist (MAACL) Hostility and Depression subscales | 1 | 0.3% | 2003 | 2003 |
|  | Negative Reactions to Care Recipient Behaviour (Kinney) | 1 | 0.3% | 2011 | 2011 |
|  | NIVEL Scale for Perceived Problems in Dementia Care (NSPP-DC) | 1 | 0.3% | 2005 | 2005 |
|  | NIVEL Scale for Perceived Problems with Specific Behaviors of patients with dementia (NSPP-SB) | 1 | 0.3% | 2005 | 2005 |
|  | Organization and Stress Scale | 1 | 0.3% | 2005 | 2005 |
|  | Philadelphia Geriatric Centre Affect Rating Scale | 1 | 0.3% | 2008 | 2008 |
|  | Spielberger State-Trait Anger Inventory | 1 | 0.3% | 2010 | 2010 |
|  | Strains in Nursing Care scale | 1 | 0.3% | 2007 | 2007 |
|  | Stress Appraisal Measure | 1 | 0.3% | 2005 | 2005 |
|  | Whitlatch Burden Interview | 1 | 0.3% | 2003 | 2003 |
|  | Zarit stress scale | 1 | 0.3% | 1999 | 1999 |
| Mastery | Unspecified self-efficacy and coping measure | 22 | 6.1% | - | - |
|  | Unspecified knowledge and mastery measure | 20 | 5.5% | - | - |
|  | Unspecified intervention satisfaction measure | 17 | 4.7% | - | - |
|  | Sense of Competence Questionnaire | 12 | 3.3% | 2000 | 2011 |
|  | Brief Coping Orientation for Problems Experienced (COPE) | 6 | 1.7% | 2006 | 2011 |
|  | Revised Scale for Caregiving Self Efficacy | 6 | 1.7% | 2003 | 2011 |
|  | Ways of coping scale | 6 | 1.7% | 1999 | 2007 |
|  | Unspecified confidence and self-esteem measure | 5 | 1.4% | - | - |
|  | Alzheimer’s Disease Caregiver Preference Questionnaire | 3 | 0.8% | 2007 | 2011 |
|  | Alzheimer's Disease Knowledge Test | 3 | 0.8% | 1994 | 2008 |
|  | Beliefs about Caregiving Scale | 3 | 0.8% | 2001 | 2005 |
|  | Caregiver self-efficacy (Pearlin) | 3 | 0.8% | 1997 | 2012 |
|  | Carers' Assessment of Managing Index | 2 | 0.6% | 2005 | 2011 |
|  | General self-efficacy scale | 2 | 0.6% | 2005 | 2010 |
|  | Jalowiec Coping Scale | 2 | 0.6% | 2005 | 2006 |
|  | Philadelphia Geriatric Center Caregiving Appraisal Scale | 2 | 0.6% | 2002 | 2009 |
|  | Symptom Management | 2 | 0.6% | 2009 | 2009 |
|  | Task Management Strategy Index | 2 | 0.6% | 2003 | 2008 |
|  | Agitation Management Self-efficacy Scale | 1 | 0.3% | 2003 | 2003 |
|  | Care Effectiveness Scale | 1 | 0.3% | 2006 | 2006 |
|  | Caregiving Mastery Index | 1 | 0.3% | 2003 | 2003 |
|  | Carers of Older People in Europe Index (COPE-Index) | 1 | 0.3% | 2011 | 2011 |
|  | Coping Resources Inventory | 1 | 0.3% | 2004 | 2004 |
|  | Coping Responses Inventory | 1 | 0.3% | 2010 | 2010 |
|  | Coping Strategies Inventory-Revised | 1 | 0.3% | 2000 | 2000 |
|  | Dementia Management Strategies Scale | 1 | 0.3% | 2008 | 2008 |
|  | Environmental Mastery scale | 1 | 0.3% | 2010 | 2010 |
|  | Expectation of Benefit Index | 1 | 0.3% | 2011 | 2011 |
|  | Geriatric Center Morale Scale | 1 | 0.3% | 2000 | 2000 |
|  | Goal Attainment Scale | 1 | 0.3% | 2011 | 2011 |
|  | Health Specific Family Coping Index | 1 | 0.3% | 1989 | 1989 |
|  | Indices of coping | 1 | 0.3% | 1999 | 1999 |
|  | Inventory of Geriatric Nursing Self-Efficacy | 1 | 0.3% | 2003 | 2003 |
|  | Knowledge of Alzheimer's Test | 1 | 0.3% | 1999 | 1999 |
|  | Knowledge of Services scale | 1 | 0.3% | 2011 | 2011 |
|  | Life-Events and difficulties | 1 | 0.3% | 2003 | 2003 |
|  | Locus of Control Scale | 1 | 0.3% | 2006 | 2006 |
|  | MacArthur Competency Assessment Tool for Clinical Research (MacCAT-CR) | 1 | 0.3% | 2006 | 2006 |
|  | Management of meaning (Pearlin) | 1 | 0.3% | 2010 | 2010 |
|  | Penn State Health Care-giving Questionnaire | 1 | 0.3% | 2009 | 2009 |
|  | Planning for Future Care Needs scale | 1 | 0.3% | 2011 | 2011 |
|  | Preparedness for Caregiving scale | 1 | 0.3% | 2011 | 2011 |
|  | Psychosocial Adjustment to Relatives Illness scale | 1 | 0.3% | 1999 | 1999 |
|  | Saunders and Courtney confidence-in-decision making | 1 | 0.3% | 1995 | 1995 |
|  | Steinmetz Control Scale | 1 | 0.3% | 2003 | 2003 |
|  | The Self-Efficacy of Dementia Care | 1 | 0.3% | 2007 | 2007 |
|  | Therapeutic engagement index | 1 | 0.3% | 2003 | 2003 |
| Mood | Center for Epidemiologic Studies Depression Scale (CES-D) | 57 | 15.8% | 1989 | 2012 |
|  | General Health Questionnaire (GHQ) | 31 | 8.6% | 1987 | 2011 |
|  | Neuropsychiatric Inventory-Distress (NPI-D) | 30 | 8.3% | 2000 | 2011 |
|  | Geriatric Depression Scale | 19 | 5.3% | 1995 | 2011 |
|  | Beck Depression Inventory (BDI) | 17 | 4.7% | 1998 | 2010 |
|  | Neuropsychiatric Inventory-Questionnaire (NPI-Q) | 12 | 3.3% | 2006 | 2011 |
|  | Brief Symptom Inventory | 8 | 2.2% | 1994 | 2007 |
|  | Hamilton Depression Scale | 8 | 2.2% | 1989 | 2010 |
|  | Pittsburgh Sleep quality Index | 8 | 2.2% | 2002 | 2011 |
|  | Unspecified satisfaction with quality of care | 8 | 2.2% | - | - |
|  | Hospital Anxiety and Depression Scale | 7 | 1.9% | 1998 | 2011 |
|  | State-Trait Anxiety Inventory | 7 | 1.9% | 1991 | 2010 |
|  | Unspecified depression | 7 | 1.9% | - | - |
|  | Unspecified satisfaction and positive states of mind | 7 | 1.9% | - | - |
|  | Positive and Negative Affect Scale (PANAS) | 5 | 1.4% | 1998 | 2011 |
|  | Hopkins Symptoms Checklist | 4 | 1.1% | 1996 | 2009 |
|  | Positive Aspects of Caregiving | 4 | 1.1% | 1997 | 2012 |
|  | Unspecified life satisfaction | 4 | 1.1% | - | - |
|  | Unspecified sleep | 4 | 1.1% | - | - |
|  | Caregiver Satisfaction Survey | 3 | 0.8% | 1997 | 2004 |
|  | Montgomery-Asberg Depression Rating Scale | 3 | 0.8% | 2002 | 2010 |
|  | Patient Health Questionnaire | 3 | 0.8% | 2003 | 2008 |
|  | Unspecified distress and upset | 3 | 0.8% | - | - |
|  | Comfort Assessment in Dying | 2 | 0.6% | 2009 | 2009 |
|  | Cornell Scale for Depression in Dementia | 2 | 0.6% | 2003 | 2010 |
|  | Delusions Symptoms States Inventory/States of Anxiety and Depression (DSSI) | 2 | 0.6% | 1987 | 1990 |
|  | Profile of Moods States (POMS) | 2 | 0.6% | 1999 | 2002 |
|  | SATMED-Q | 2 | 0.6% | 2009 | 2009 |
|  | Self Reporting Questionnaire-20 | 2 | 0.6% | 2007 | 2009 |
|  | Self-Assessing Depression Scale (SADS) | 2 | 0.6% | 1997 | 2009 |
|  | Unspecified grief | 2 | 0.6% | - | - |
|  | Unspecified guilt | 2 | 0.6% | - | - |
|  | Befindlichkeits-Skala | 1 | 0.3% | 2000 | 2000 |
|  | Camberwell Family Interview | 1 | 0.3% | 2002 | 2002 |
|  | Caregiver Distress About Night-time Activity | 1 | 0.3% | 2010 | 2010 |
|  | Caregiver Distress Scale | 1 | 0.3% | 2003 | 2003 |
|  | Carers’ Assessment of Satisfaction Index | 1 | 0.3% | 2008 | 2008 |
|  | Carlsson Visual Analogue Scale | 1 | 0.3% | 2011 | 2011 |
|  | Depression, Anxiety and Stress Scale (DASS-21) | 1 | 0.3% | 2008 | 2008 |
|  | Dysfunctional Thoughts about Caregiving | 1 | 0.3% | 2010 | 2010 |
|  | Epworth Sleep Questionnaire | 1 | 0.3% | 2010 | 2010 |
|  | Eysenck Personality Questionnaire revised | 1 | 0.3% | 2011 | 2011 |
|  | Family caregiver distress (Pearlin) | 1 | 0.3% | 2005 | 2005 |
|  | General Sleep Disturbance Scale | 1 | 0.3% | 2009 | 2009 |
|  | Ilfeld Psychiatric Symptoms Index | 1 | 0.3% | 2003 | 2003 |
|  | Impact of event | 1 | 0.3% | 2009 | 2009 |
|  | Inventory of Complicated Grief | 1 | 0.3% | 2003 | 2003 |
|  | Kessler distress scale | 1 | 0.3% | 2011 | 2011 |
|  | Kessler-6 | 1 | 0.3% | 2008 | 2008 |
|  | Leisure Time Satisfaction | 1 | 0.3% | 2003 | 2003 |
|  | Marwit and Meuser Caregiver Grief Inventory-Short Form (MMCGI-SF) | 1 | 0.3% | 2010 | 2010 |
|  | Mental Health Index | 1 | 0.3% | 2006 | 2006 |
|  | Morin Daily Sleep Diary | 1 | 0.3% | 2006 | 2006 |
|  | NEO Personality Inventory (NEO-PI) | 1 | 0.3% | 2011 | 2011 |
|  | Night Time Activity Worry – Scale | 1 | 0.3% | 2012 | 2012 |
|  | Ohio Department of Aging Family Satisfaction Instrument | 1 | 0.3% | 2011 | 2011 |
|  | Penn State Mental Health Questionnaire-20 | 1 | 0.3% | 1999 | 1999 |
|  | Penn State Worry Questionnaire | 1 | 0.3% | 2009 | 2009 |
|  | Poulshok and Deimling cognitive subscale | 1 | 0.3% | 2000 | 2000 |
|  | Prolonged Grief Disorder-12 | 1 | 0.3% | 2008 | 2008 |
|  | Psychological Distress Index | 1 | 0.3% | 2005 | 2005 |
|  | Satisfaction with Care at End-of-Life in Dementia | 1 | 0.3% | 2008 | 2008 |
|  | Schedule for the Evaluation of Individualized Quality of Life | 1 | 0.3% | 2006 | 2006 |
|  | State-Trait Personality Inventory | 1 | 0.3% | 2003 | 2003 |
|  | Swiss health survey | 1 | 0.3% | 2006 | 2006 |
|  | Symptom Check List 90 Revised | 1 | 0.3% | 1999 | 1999 |
|  | Taylor Manifest Anxiety Scale | 1 | 0.3% | 2002 | 2002 |
|  | Texas Revised Inventory of Grief | 1 | 0.3% | 2003 | 2003 |
|  | Zung depression scale | 1 | 0.3% | 1989 | 1989 |
| Quality of life | Short Form-36 (SF-36) | 32 | 8.9% | 2001 | 2011 |
|  | EuroQoL (EQ-5D) | 18 | 5.0% | 2001 | 2011 |
|  | Unspecified health/quality of life | 11 | 3.0% | - | - |
|  | World Health Organization Quality of Life-Bref (WHOLQOL-BREF) | 8 | 2.2% | 2007 | 2011 |
|  | Health Utilities Index(HUI) | 4 | 1.1% | 1999 | 2010 |
|  | Health Status Questionnaire | 3 | 0.8% | 2003 | 2011 |
|  | Caregiver Health and Health Behaviours (Posner) | 2 | 0.6% | 2003 | 2007 |
|  | Duke Health Profile | 2 | 0.6% | 2005 | 2010 |
|  | Nottingham Health Profile | 2 | 0.6% | 2008 | 2010 |
|  | Perceived Change Index | 2 | 0.6% | 2003 | 2010 |
|  | Philadelphia Geriatric Centre multilevel assessment | 2 | 0.5% | 1989 | 2001 |
|  | Beschwerdeliste | 1 | 0.3% | 2009 | 2009 |
|  | Caregiver Quality of Life Instrument (Mohide) | 1 | 0.3% | 1991 | 1991 |
|  | CarerQol-7D | 1 | 0.3% | 2009 | 2009 |
|  | CQOL (Logsdon) | 1 | 0.3% | 2010 | 2010 |
|  | Cumulative Illness Rating Scale | 1 | 0.3% | 2010 | 2010 |
|  | DQOL | 1 | 0.3% | 2008 | 2008 |
|  | General Medical Health Rating | 1 | 0.3% | 2003 | 2003 |
|  | General Well-being Adjustment Scale (Brook) | 1 | 0.3% | 2001 | 2001 |
|  | General Well-Being scale | 1 | 0.3% | 2003 | 2003 |
|  | General well-being schedule | 1 | 0.3% | 2011 | 2011 |
|  | Giessen physical health | 1 | 0.3% | 2005 | 2005 |
|  | Health Assessment Scale | 1 | 0.3% | 2000 | 2000 |
|  | Norderstedt | 1 | 0.3% | 2004 | 2004 |
|  | Personal Well-being Index | 1 | 0.3% | 2010 | 2010 |
|  | Physical Activity Question Score (Voorrips) | 1 | 0.3% | 2011 | 2011 |
|  | Quality of Life questionnaire (Ruiz) | 1 | 0.3% | 2002 | 2002 |
|  | Rapid Assessment of Physical Activity scale | 1 | 0.3% | 2011 | 2011 |
|  | Ruiz and Baca's Questionnaire-39 | 1 | 0.3% | 2010 | 2010 |
|  | Siegrist Profil der Lebensqualität chronisch (PLC) | 1 | 0.3% | 2005 | 2005 |
|  | Symptom Questionnaire | 1 | 0.3% | 2004 | 2004 |
|  | Well-Being Scale | 1 | 0.3% | 2001 | 2001 |
|  | World Health Organisation's Alcohol Use Disorders Identification Test | 1 | 0.3% | 2011 | 2011 |
| Social support and relationships | Unspecified social support and relationships measure | 20 | 5.5% | - | - |
|  | Social Support Questionnaire | 7 | 1.9% | 1999 | 2011 |
|  | Stokes Social Support network List | 4 | 1.1% | 2006 | 2010 |
|  | Inventory of Socially Supportive Behaviours | 3 | 0.8% | 2003 | 2011 |
|  | Multidimensional scale of perceived social support | 3 | 0.8% | 2008 | 2011 |
|  | Quality of the carer-patient relationship | 3 | 0.8% | 2009 | 2011 |
|  | Unspecified loneliness measure | 3 | 0.8% | - | - |
|  | Duke Social support questionnaire | 2 | 0.6% | 1999 | 2010 |
|  | Perceived Social Support scale (Pearlin) | 2 | 0.6% | 2002 | 2002 |
|  | Practitioner Assessment of Network Type | 2 | 0.6% | 2008 | 2011 |
|  | Social Provisions Scale | 2 | 0.6% | 2002 | 2011 |
|  | Clifton Assessment Procedures for Elderly Behavioural Rating Scale (CAPE-BRS) | 1 | 0.3% | 1999 | 1999 |
|  | Family APGAR questionnaire | 1 | 0.3% | 2010 | 2010 |
|  | Family Involvement scale | 1 | 0.3% | 2007 | 2007 |
|  | Family Support Services Index | 1 | 0.3% | 2011 | 2011 |
|  | Interpersonal Social Evaluation List | 1 | 0.3% | 2002 | 2002 |
|  | Lubben Social Network Index | 1 | 0.3% | 2003 | 2003 |
|  | Marital Needs Satisfaction Scale | 1 | 0.3% | 2000 | 2000 |
|  | Modified Social Support (Barrera) | 1 | 0.3% | 2003 | 2003 |
|  | Moses | 1 | 0.3% | 2008 | 2008 |
|  | Mutual Communal behaviours Scale | 1 | 0.3% | 2008 | 2008 |
|  | Mutuality Scale of the Family Care Inventory | 1 | 0.3% | 2011 | 2011 |
|  | Personal Resource Questionnaire (PRQ-85) | 1 | 0.3% | 2004 | 2004 |
|  | Positive and Negative Social Exchanges | 1 | 0.3% | 2011 | 2011 |
|  | Received Social Support scale | 1 | 0.3% | 2007 | 2007 |
|  | Relationship Attribution Measure | 1 | 0.3% | 2005 | 2005 |
|  | Sherbourne Social Support | 1 | 0.3% | 2007 | 2007 |
|  | Systematic Care Program for Dementia | 1 | 0.3% | 2009 | 2009 |
|  | Thomas Assessment of communication inadequacy | 1 | 0.3% | 2001 | 2001 |
| Staff competency and morale | Maslach Burnout Inventory | 10 | 2.6% | 2004 | 2011 |
|  | Unspecified job satisfaction and competence measure | 7 | 1.8% | - | - |
|  | Approaches to Dementia Questionnaire | 4 | 1.1% | 2005 | 2012 |
|  | Modified Nursing Care Assessment scale M-NCAS | 3 | 0.8% | 2004 | 2011 |
|  | Blenkner (relationship between nurses and person with dementia) | 1 | 0.3% | 2004 | 2004 |
|  | Canadian occupational performance measure | 1 | 0.3% | 2011 | 2011 |
|  | Creative Climate Questionnaire | 1 | 0.3% | 1994 | 1994 |
|  | Experience and Assessment of work (VBBA) | 1 | 0.3% | 2011 | 2011 |
|  | Family behaviours and empathy scale | 1 | 0.3% | 2007 | 2007 |
|  | Formal Caregivers’ Attitude toward Feeding Dementia Patient Questionnaire | 1 | 0.3% | 2005 | 2005 |
|  | Formal Caregivers’ Behaviours toward Feeding Dementia Patients Observation Checklist | 1 | 0.3% | 2005 | 2005 |
|  | Formal Caregivers’ Knowledge of Feeding Dementia Patient Questionnaire | 1 | 0.3% | 2005 | 2005 |
|  | Iowa Dependent Adult Abuse Nursing Home Questionnaire | 1 | 0.3% | 2010 | 2010 |
|  | Kazdin's Treatment Evaluation Inventory | 1 | 0.3% | 1995 | 1995 |
|  | Knowledge and Management of Abuse | 1 | 0.3% | 2010 | 2010 |
|  | Leiden Quality of Work Questionnaire | 1 | 0.3% | 2011 | 2011 |
|  | Maastricht Work Satisfaction Scale for Healthcare | 1 | 0.3% | 2005 | 2005 |
|  | Sense of Competence in Dementia care Staff (SCIDS) | 1 | 0.3% | 2012 | 2012 |
|  | STAR Staff Feedback Questionnaire (SSFQ) | 1 | 0.3% | 2012 | 2012 |
|  | Stress screening of human services (nurses' occupational stress) | 1 | 0.3% | 2009 | 2009 |
|  | Subjective work pressure (Potentialanalyse stationärer Altenpflege - PASTA) | 1 | 0.3% | 2010 | 2010 |
|  | Work Stress Inventory | 1 | 0.3% | 2005 | 2005 |
